# The overlap between miscarriage and extreme preterm birth in a limited-resource setting on the Thailand-Myanmar border: a population cohort study

**DOI:** 10.12688/wellcomeopenres.10352.3

**Published:** 2018-12-06

**Authors:** Rose McGready, Moo Kho Paw, Jacher Wiladphaingern, Aung Myat Min, Verena I. Carrara, Kerryn A. Moore, Sasithon Pukrittayakamee, François H. Nosten

**Affiliations:** 1 Shoklo Malaria Research Unit, Mahidol-Oxford Tropical Medicine Research Unit, Mahidol University, Mae Sot, Thailand; 2 Centre for Tropical Medicine and Global health, Nuffield Department of Medicine Research Building, University of Oxford, Oxford, UK; 3 Department of Medicine, Swiss Tropical and Public Health Institute, 4002 Basel, Switzerland; 4 Centre for Epidemiology and Biostatistics, Melbourne School of Population and Global Health, University of Melbourne, Melbourne, Australia; 5 Macfarlane Burnet Institute for Medical Research and Public Health, Melbourne, Australia; 6 Mahidol-Oxford Tropical Medicine Research Unit, Mahidol University, Bangkok, Thailand

**Keywords:** extreme preterm birth, limited-resource, low-income, marginalized, miscarriage, neonatal death, stillbirth, ultrasound

## Abstract

**Background** *: *No universal  demarcation of gestational age  distinguishes miscarriage and stillbirth or extreme preterm birth (exPTB). This study provides a synopsis of outcome between 22 to <28 weeks gestation from a low resource setting.

**Methods** *: *A retrospective record review of a population on the Thailand-Myanmar border was conducted. Outcomes were classified as miscarriage, late expulsion of products between 22 to < 28 weeks gestation with evidence of non-viability (mostly ultrasound absent fetal heart beat) prior to 22 weeks; or  exPTB (stillbirth/live born) between 22 to < 28 weeks gestation when the fetus was viable at ≥22 weeks. Termination of pregnancy and gestational trophoblastic disease were excluded.

**Results** *: *From 1995-2015, 80.9% (50,046/ 61,829) of registered women had a known pregnancy outcome, of whom 99.8% (49,931) had a known gestational age. Delivery  between 22 to <28 weeks gestation included 0.9% (472/49,931) of pregnancies after removing 18 cases (3.8%) who met an exclusion criteria. Most  pregnancies had an ultrasound: 72.5% (n=329/454);  43.6% (n=197) were classified as  miscarriage and 56.4% (n=257) exPTB.  Individual record review of miscarriages estimated that fetal death had occurred at a median of 16 weeks, despite late expulsion between 22 to <28 weeks. With available data (n=252, 5 missing) the proportion of stillbirth was 47.6% (n=120), congenital abnormality 10.5% (24/228, 29 missing) and neonatal death was 98.5% (128/131, 1 missing). Introduction of ultrasound was associated with a 2-times higher odds of classification of outcome as exPTB rather than miscarriage.

**Conclusion** *: *In this low resource setting few (<1%) pregnancy outcomes occurred in the 22 to <28 weeks gestational window; four in ten  were miscarriage (late expulsion) and neonatal mortality approached 100%.  In the scale-up to preventable newborns deaths (at least initially) greater benefits will be obtained by focusing on the viable newborns of ≥ 28 weeks gestation.

## Introduction

To determine progress towards the Sustainable Developmental Goal (SDG) 3.2 “by 2030, to end preventable deaths of newborns and children under 5 years of age”, standardized data collection is critical ^1– 3^. Without a universally accepted definition to distinguish fetal death from miscarriage (spontaneous abortion) versus stillbirth ^4, 5^ ( Supplementary File 1) and the wide variation in standard of care available for live born extreme preterm births (exPTB), individual patient care and assessment of SDG progress are affected ^6^. Miscarriage remains an awkward condition to define due to variability between and even within countries particularly in relation to laws surrounding termination of pregnancy ^7^. There are obvious legal, religious and cultural sensitivities to pregnancy termination which may inhibit large organizations from specifying an upper limit to define miscarriage ^8^. The International Classification of Diseases, provides a graded definition of miscarriage (pregnancy loss before 22 completed weeks gestation); and stillbirth (expulsion of a fetus with no signs of life) classified as early fetal death (of a fetus weighing 500 g or more, or aged 22 weeks or more, or with a body length of 25 cm or more); late fetal death (of a fetus of 1000 g or more, or aged 28 weeks or more, or with a body length of 35 cm or more) ^9^. These definitions overlap. For example, is a stillborn 1000 g fetus with accurately determined gestation born at 27 ^+2^ weeks ^+days^, a late fetal death because of the birth weight or an early fetal death because of the gestation? For organizations that utilize the WHO definition of stillbirth recommended for global comparison i.e.: “a baby born with no signs of life at or after 28 weeks gestation” ^10^ confusion arises with live newborns before 28 weeks. It is difficult to explain to health care workers that **still**birth starts at 28 weeks but **live** birth can start at <28 weeks.

On the Thailand-Myanmar border at Shoklo Malaria Research Unit (SMRU), the WHO definition of stillbirth based upon reaching 28 weeks gestation has been used for the past 32 years ( Supplementary File 1). Based on this definition there has been a significant decline in stillbirth (28 to 14 per 1,000 live births) and neonatal mortality (49 to 11 per 1,000 livebirths) from 1993–1996 to 2008–2011 ^11, 12^. Miscarriage rates have been stable over time at 10% (2257/23 118) from 1994 to 2013 ^13^. The outcomes of pregnancies of 28 weeks or more gestation in this area have been published previously and are not the focus of this analysis ^12, 14, 15^.

One of the most significant factors in this border region has been the 3 decades long struggle with recurring loss of antimalarials to treat patients with multi-drug resistant *P. falciparum* malaria ^16^. The Thailand-Myanmar border was one of the first places in the world to introduce artemisinin-based combination therapy for treatment of malaria in the general population ^17^. The artemisinin derivatives were also used initially for treatment of malaria in the 2 ^nd^ and 3 ^rd^ trimesters of pregnancy or to save the life of the mother with severe malaria in any trimester ^18^. This class of drug distinguished itself in pregnancy as it was first reported by Chinese scientists to cause fetal resorption (or early miscarriage) ^19^ so it was important to be able to determine gestational age during pregnancy and to have a clear definition of trimester and miscarriage. Quinine remained the WHO recommended drug for women in the first trimester of pregnancy and systematic data collection by local health workers has built an evidence base that has made a significant contribution to global knowledge about antimalarial drug use in pregnancy particularly in the first trimester ^13, 20^.

In the 1990’s at SMRU, gestational age during pregnancy was estimated from fundal height by a formula developed for the study population ^21, 22^ or by last menstrual period (LMP), and from late 2001 by ultrasound ^23^. In 1995 the only tools available in the field were pregnancy tests, symphysis fundal height measurement (SFH), pinnard and at best (late 1990’s) a small hand-held Doppler to detect fetal heart beat. SFH measurements were routinely carried out at antenatal care and quality control measurement exercises were conducted because it was so heavily relied upon. SFH was examined at every visit until the fundus of the uterus could be felt for the first time and thereafter approximately every 2–4 weeks unless more frequent repeated measures were indicated. In these circumstances and in the absence of bleeding or fetal movements, detection of loss of fetal viability was identified late. When ultrasound was introduced in the area, a first trimester or first ANC visit scan was followed up by a repeat scan initially at 18 weeks and later shifted to 22 weeks. Gestational age of a non-viable fetus could be more readily obtained by ultrasound but for SMRU data to be consistent with the former data, the end of gestation of pregnancy has remained constant, as the date of expulsion of the products of conception.

At the same time, work has been standardized for local health workers by use of obstetric guidelines ^24^. In the guidelines the working definition of miscarriage is outcome of pregnancy before 28 weeks, while births (stillbirth and live birth) commence from 28 weeks, as does reporting of neonatal deaths. This definition has remained unchanged with the establishment of a special care baby unit in this resource limited setting where assisted ventilation of newborns is not available (i.e birth before 28 weeks is not viable) ^12^.

The objective of this study is to bring clarity to the nature of the products of conception that are expulsed in the 22 to <28 weeks gestational age window in a low resource setting, highlighting operational issues and the role of ultrasound ^9^.

## Methods

### Setting

**Figure 1.**
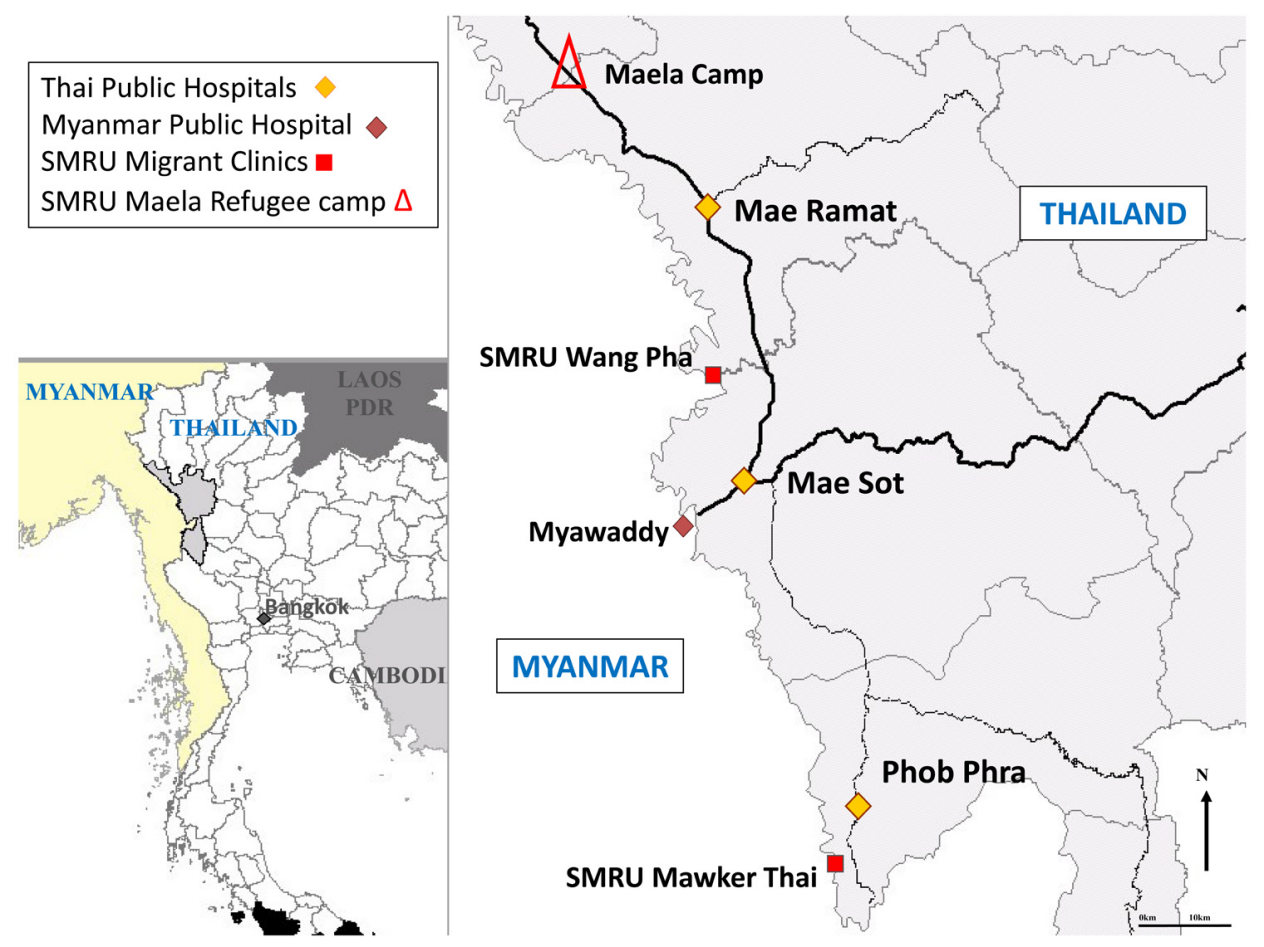
Map of the border area. Location of SMRU clinics where pregnant women can attend for antenatal care and childbirth.

SMRU is an operational field-based research unit uniquely combining humanitarian work with research of direct relevance to the local population. It is a limited-resource setting working with marginalized populations on the western border of Thailand in Tak Province. In this area, there are an estimated 140,000 refugees and 200,000 migrants from Myanmar. There have been decades of neglect of the health system in Myanmar and the government is currently trying to address this. The refugee situation on the border of Thailand and Myanmar is amongst the most protracted in Asia but it has set a scenario of how health, particularly among pregnant women and obstetric emergencies of Myanmar people are managed. Due to conflict in Eastern Kayin state, refugees obtained surgical care in Thailand hospitals via a system of referral from Community-Based- and Non-Government Organizations. Health care for migrant pregnant women was established in 1998 by SMRU as there were minimal services available for them ( Figure 1).

At SMRU, place and attendance at birth has shifted from 75% occurring at home with traditional birth attendants with no formal training in 1986, to more than 80% of births occurring in health facilities with skilled attendants in 2015 ^15^. SMRU is staffed predominantly by locally trained workers for antenatal care and ultrasound ^23^, child birth ^15, 24^ and emergencies in adults and neonates ^12^. Local medics, midwives and nurses do the majority of the clinical work and expatriate doctors assist local staff with 24-hour back up. Ultrasound quality has been measured previously in this setting ^23^ and the eight sonographers undergo a small quality control every 6 months by a clinician (5 scans, blinded to gestation, to check for image quality and intra and inter-observer error) ^24^. Over 3,000 women register at SMRU antenatal clinics annually and these were well established before birth services were offered. The first birthing unit was opened at Shoklo refugee camp in 1986, with border skirmishes and closure of Shoklo, this was relocated to Maela Refugee camp in 1995, and two more units for marginalized migrant workers were opened in Wang Pha in Dec-2007 and in Maw Ker Thai in April 2010 ( Figure 1).

The seven signal functions for Basic Emergency Obstetric and Newborn Care, including parenteral administration of an oxytocic, antibiotics and anticonvulsants, removal of retained products of conception, assisted vaginal birth including breech birth, resuscitation of the newborn using a bag and mask for infants ≥ 28 weeks gestation, and screened blood transfusions, are provided by local staff. A description of the special care baby unit for neonates has been detailed by Turner *et al.* ^12^, but there is no capacity for intubation and assisted ventilation and prohibitive costs limit newborn referrals. Aminophylline is used in place of caffeine for apneas, again due to costs. Local protocols guide care and are regularly updated with trainings of the local staff. The protocol for preterm labour recommends dexamethasone (betamethasone is not available) and nifedipine at a gestation of at least 28 weeks. If extreme PTB (exPTB) occurs (<28 weeks) infants are provided with palliative care. Parents are involved and counseled in the process ^12^. No resuscitative efforts are offered to live born infants of 22 to <28 weeks gestation as no further sophisticated care i.e. intubation, incubators, surfactant, parenteral nutrition etc. is available ^25^. Women who need caesarean section or who have complex medical conditions are transferred by car to Thai hospitals (45 to 90 minutes away).

### Pregnancy ultrasound and fetal viability

Ultrasound scans have been performed by local health workers using various scanners, including Toshiba Powervision 7000, Dynamic Imaging (since 2001), Fukuda Denshi UF 4100, and General Electric Voluson-1. Since 2001 all women have been offered two scans: once at booking to determine viability, number of fetus and gestation, regardless of how far progressed the pregnancy is, but preferably between 8 and 14 weeks; and again at 22 (18–24) weeks to reassess viability, measure fetal biometry and major abnormalities and determine placental location ^23^. Ultrasound can be repeated at any time as required. For example, if a woman reported absence of fetal movement, or bleeding, or the fundal height did not increase, an ultrasound could be done to determine viability. Measurement of the fetus size at each scan was encouraged. Loss of fetal heart beat could also be an incidental finding when the woman attended for her second scan. Loss of viability with ultrasound could be confirmed by presence of a fetus and absent fetal heartbeat. In some cases, ultrasound confirmed pregnancies persisting to 22 weeks gestation, but a fetus was never observed, e.g. anovulatory gestation or non-classic gestational trophoblastic disease.

In this setting SFH measurement has been important to determine whether a woman was in the first or second trimester which made the difference between being able to use quinine or artesunate to treat uncomplicated malaria. At antenatal care, pregnant women had abdominal palpation at each visit until the SFH was first palpable and measurable above the symphysis, then SFH was checked monthly up to 32 weeks and weekly from 36 weeks gestation. In case of doubt, urine (sometimes serum) pregnancy testing was available. It was and still is common to have the SFH measured multiple times in a single pregnancy ^21, 26^. Before ultrasound availability, loss of fetal viability could be confirmed by SFH measurements, which were increasing and then levelled off, or decreased with unexpected SFH results being confirmed by a clinician), complaints of loss of fetal movement or never feeling movement, bleeding episodes or expulsion of products. Quality control exercises of SFH involved comparing 20 women per month between SFH measurers – a difference of > 1cm was considered inacceptable and corrections of the firmness of applying the tape measure, correctly placing the end of the tape measure on the upper border of the symphysis pubis and identifying the fundus, were made during these sessions.

Management of fetal loss diverged from high income settings. Before misoprostol was available, induction of pregnancies with confirmed fetal loss was difficult and if there was no vaginal bleeding and the cervix was not open a conservative management style was adopted. Induction with syntocinon, the only available agent for many years, was frequently a prolonged and unsuccessful process. In this low resource context, only surgical emergencies were referred to tertiary hospitals, so non-viable pregnancy loss, with no imminent danger signs did not qualify for referral.

### Data extraction and data definitions

All birth records at SMRU are computer based and paper-based records are archived at SMRU head office in Mae Sot. The original paper records initially matched data collection used by Médecins Sans Frontières (MSF) in humanitarian settings. In 1998 computer based registration commenced with adjustments to the MSF form, in late 2001 ultrasound commenced requiring further amendments, and in late 2007 all data was captured in real time in an application developed by Technology Sans Frontières (TSF) with data limitations set to alert users to inaccurate entry. Verification of paper and computer based data has taken place over decades to a computer based record system.

All pregnancy records from 1995 until 2015 in the window period from 22 to <28 weeks gestation were selected and reviewed case by case by for the present study. The starting point of 1995 was selected, since the first local guideline for obstetrics was introduced at this time.

For each record the following evaluation was conducted using a step-wise query process:

- a) Was there evidence of in utero fetal demise (no FHB) before 22 weeks? If yes, could the gestation of loss be estimated? For example, was fetal anthropometry measured by ultrasound when absence of fetal heart beat was confirmed; or did the SFH measurements stall or decrease (and at how many centimeters) before fetal heart beat could be heard or fetal movements felt, or did the mother report that she never felt fetal movements? Was there evidence that there was never a fetus i.e. that the pregnancy was never viable? Did ultrasound measure only annovulatory pregnancy (blighted ovum)?
- b) Was there evidence of in utero fetal viability at 22 to <28 weeks gestation (FHB by ultrasound, hand held Doppler or Pinnard)? If yes, what was the estimated gestation at loss of viability and were there any signs of life (clinic births-heart beat by auscultation and home births-respiratory effort/movement) at birth?
- c) If the outcome was a live birth, what was the neonatal outcome?

Records with evidence of in utero fetal demise before 22 weeks but expulsion between 22 to <28 weeks were classified as miscarriage (late expulsion). Pregnancies with evidence of fetal viability at ≥22 weeks that were expulsed between 22 to <28 weeks were classified as exPTB (live or stillborn). Neonatal mortality was defined as death in the first 28 days of a live born neonate of 22 to <28 weeks gestation. Gestational trophoblastic disease and termination of pregnancy were excluded. Congenital abnormalities were coded using the ICD-10 criteria ^9^.

### Statistical analysis

Gestation was reported by week, for example 22 weeks included women from 22 ^+0^ to 22 ^+6^ weeks, (i.e 22 weeks plus 6 days) of pregnancy. Continuous normally distributed data, such as gestation and birth weight, were described using the mean, and standard deviation (SD) and compared with the Student’s t-test. Only the first born twin birth weight was retrieved from the electronic files. Non-parametric data, such as gravidity, were described using median and 25 ^th^-75 ^th^ percentiles and compared with the Mann-Whitney U test. Proportions were compared using the Chi-squared test. To assess the role of ultrasound in the final classification as exPTB rather than miscarriage between 22 to <28 weeks gestation, univariable and multivariable logistic regression was used to determine the association between ultrasound use and outcome, adjusted for first ANC attendance in first trimester and delivering with a skilled attendant (confounders identified *a priori*). Data was analysed using SPSS version 20 (IBM SPSS, Armonk, NY, USA) and Stata version 13 (StatCorp, College Station, TX, USA).

### Ethics statement

Ethical approval for retrospective analysis of pregnancy records was given by the Oxford Tropical Research Ethics Committee (OXTREC 28–09) and after discussion with the local Community Advisory Board (TCAB-4/1/2015)

## Results

**Figure 2.**
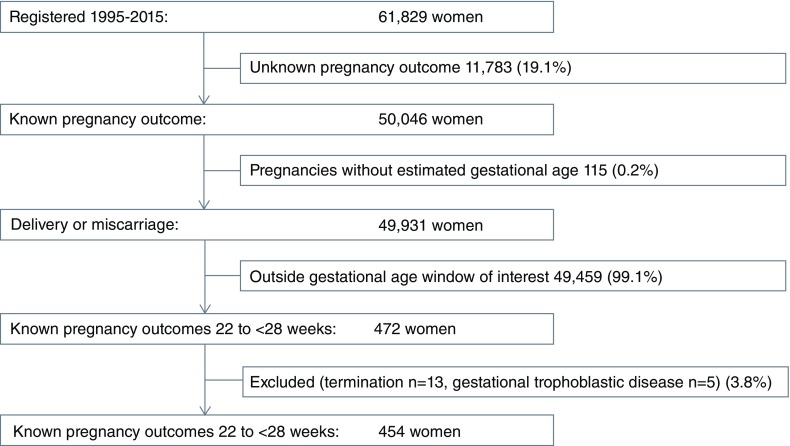
Study flow. Selection of women in the cohort of 22 to < 28 weeks gestation.

Between 1995 and 2015, 80.9% (50,046/61,829) of women registered to antenatal care had a known pregnancy outcome and were included in the present study. Only a small proportion, 0.2% (115/50,046), of these pregnancies could not be assigned a reliable gestational age ( Figure 2). The proportion of all pregnancy outcomes within the gestational window of 22 to <28 weeks was small: 0.9% (472/49,931) and most of these had an obstetric ultrasound scan and dating: 73.1% (345/472).

There were 3.8% (18/372) excluded from analysis: termination of pregnancy involved 13 cases including: six ultrasound confirmed major fetal abnormality (five anencephalic, one holoprosencephaly), two life-threatening maternal conditions both with uncontrollable severe pre-eclampsia, and five self-induced (one of whom was recently widowed); and five gestational trophoblastic disease. The demographic characteristics of the remaining 454 pregnancy outcomes are summarized in Table 1. The majority of these pregnancies were dated by ultrasound: 72.5% (329/454). The numbers and proportions of pregnancy outcome for each gestational age week from 22 to <28 are shown in Table 2. There were 6.2% (28/454) twin pregnancies.

**Table 1.** Baseline demographic characteristics of 454 women with pregnancy outcome 22 to <28 weeks gestation. *Missing data: weight first ANC, weight less than 40 kg at first ANC n=3; BMI and BMI category n=158; Anemia at first ANC visit n=24. Abbreviation: ANC, antenatal clinic.

Characteristic	Value
Age, years, mean [±SD], [min-max]	28 [±8] [13–48]
Gravidity, median {25 ^th^-75 ^th^ percentile}, [min-max]	3 {2–5},[1–15]
Parity, median {25 ^th^-75 ^th^ percentile}, [min-max]	2 {0–4},[0–11]
Primigravida, % (n)	24.4 (111/454)
Grandmultipara (more than 4 births), % (n)	16.5 (75/454)
Weight first ANC, kg, mean [±SD], [min-max] *	48 [±8] [31–81]
Weight less than 40 kg first ANC, n (%)	8.9 (40/451)
BMI, kg/m ^2^ at first ANC *, mean [±SD] [min-max];	21.5 [±3.3], [13.6–34.2]
Underweight (<18.5), % (n)	14.2 (42)
Normal weight (18.5 to < 23), % (n)	61.7 (182)
Over weight (23 to <27.5), % (n)	18.0 (53)
Obese (≥ 27.5), % (n)	6.1 (18)
Number of ANC visits, median{25 ^th^-75 ^th^ percentile}, [min-max]	6 {3-11}, [1-22]
A total of 4 or more ANC visits, % (n)	58.4 (265/454)
Anemia at first ANC, % (n) *	12.3 (53/430)
First ANC visit in trimester one (less than 14 weeks), % (n)	55.5 (252/454)

**Table 2.** Numbers and proportions of pregnancy outcomes by gestational age week 22 to <28 weeks.

		Weeks gestation at pregnancy outcome					
	Total	22	23	24	25	26	27
N	454	71	78	80	60	86	79
Miscarriage	197 (43.6)	55 (77.5)	47 (60.3)	41 (51.3)	20 (33.3)	15 (17.4)	19 (24.1)
Extreme PTB	257 (56.4)	16 (22.5)	31 (39.7)	39 (48.8)	40 (66.7)	71 (82.6)	60 (75.9)
Twins ^a^	28 (61.8)	1 (1.4)	5 (6.4)	2 (2.5)	7 (11.7)	6 (7.0)	7 (8.9)
*Extreme PTB*							
Missing data on live and still birth	5	0	0	1	2	1	1
Stillbirth	120/252 (47.6)	12 (75.0)	22 (71.0)	18 (47.4)	18 (47.4)	33 (46.1)	17 (28.8)
Live birth	132/252 (52.4)	4 (25.0)	9 (29.0)	20 (52.6)	20 (52.6)	37 (52.9)	42 (71.2)
*Survival of Newborns*							
Missing data NND	1 ^a^	0	0	0	0	0	1
NND day 1	87/131 (66.9)	4 (100.0)	9 ^c^ (100.0)	17 (85.0)	18 (90.0)	22 (59.5)	18 (42.9)
NND day 3	114/131 (87.0)	4 (100.0)	9 ^c^ (100.0)	19 (95.0)	19 (95.0)	31 (83.8)	33 (78.6)
NND day 28	129/131 (98.5)	4 (100.0)	9 (100.0)	20 (100.0)	20 (100.0)	37 (100.0)	39 (95.2)
Alive > 1 month	2	0	0	0	0	0	2 ^d^

Data are n (%) unless otherwise stated; Abbreviations: n.a, NND neonatal death; PTB preterm birth

^a^Most twins pregnancies were extreme PTB except for 2 which were miscarriage at 23 weeks

^b^Born alive and probably died but data not available in the record

^c^not sure exact days NND (only 700g and born at home, brought to clinic)

^d^One died at day 33; one was still alive at 40 months of age

### Miscarriage

Of the 454 pregnancy outcomes from 22 to <28 weeks gestation 197 (43.5%) were miscarriage (loss of viability before 22 weeks) two of which were twin gestations (23 weeks) ( Table 2). More than half of 197 miscarriages occurred at 22 and 23 weeks gestation 27.9% (55) and 23.9% (47); with 24, 25, 26, and 27 weeks accounting for 20.8% (41), 10.2% (20), 7.6% (15) and 9.6% (19), respectively.

Loss of viability was not possible to determine for 11 records (nine indicated spontaneous miscarriage and two records could not be located) leaving 186 records. Evidence of fetal demise (absence of fetal heart beat) also known as fetal death in utero, occurred in 60.2% (112/186), with most of these determined by ultrasound ( Table 3). The proportion with stalling or decreases in SFH was higher before ultrasound was introduced ( Table 3). Annovulatory pregnancy (blighted ovum) was also a reason for expulsion of products of pregnancy from 22 to < 28 weeks of pregnancy in this setting: 16.8% (19/113) when ultrasound was available ( Table 3). In the 112 cases with fetal death in utero the estimated time from non-viability to expulsion was a median of 7 ^+1^ [IQR 4 ^+5^ to 11 ^+0^] weeks ^+days^. ( Table 3), and not significantly different pre and post ultrasound: 6 ^+3^ (n=24) vs 7 ^+4^ weeks ^+days^ (n=88), p=0.084.

**Table 3.** Reason for late miscarriage (expulsion 22 to <28 weeks gestation) and the estimated gestational age of loss of viability (median [min-max] in weeks).

	Before ultrasound (n=73)		Ultrasound (n=113)	
Event	N (%)	EGA of loss of viability	N (%)	EGA at loss of viability
Fetal death in utero	24 (32.9)	16 ^+6^ [11 ^+1^-21 ^+6^]	88 (77.9)	16 ^+3^ [7 ^+2^-21 ^+6^]
SFH stalled or decreased	41 (56.2)	16 ^+0^ [8 ^+0^-21 ^+0^]	6 (5.3)	16 ^+0^ [10 ^+0^-17 ^+2^]
Annovulatory	8 (10.9)	8 ^+0^ [ ^7+0^-8 ^+0^]	19 (16.8)	8 ^+0^ [7 ^+0^-12 ^+0^]

### Extreme preterm birth (22 to <28 weeks)

There were 56.4% (257/454) of women with an exPTB of which 89.9% (231/257) were singletons and 10.1% (26/257) were twins ( Table 2). The gender of the infant was missing for 30.0% (77/257) cases, with the remainder including 56.1% (101/180) males and 43.9% (79/180) females.

Amongst the 257 pregnancies ending in exPTB, 1.9% (5/257) had missing data on whether the infant was still- or live born ( Table 2), and for the remaining cases, 47.6% (120/252) were recorded as stillbirths (including first born twin), and 52.4% (132/252) were born alive. Most women birthed vaginally, 98.0% (253/257), with 66.5% (171/257) in SMRU clinic. There were 1.6% (4/252) delivered by caesarean sections. These four cases were in singleton pregnancies, three at 27 weeks and one at 26 weeks, three of whom had placental pathologies (two placenta praevia and one placental abruption) and one with preterm labour and transverse presentation. These four births all ended in stillbirth with a birth weight available for one case (900g).

The birth weight measured in singletons was not available for 42.0% (108/257) of neonates. For the 75 homebirths this is not surprising. Birth weight of 17 congenitally abnormal infants was excluded from analysis. Birth weight in live born, normal singletons in the period before ultrasound (n=18) and when ultrasound was available (n=67) was similar: mean±SD (range): 817±253 (350–1300) and 875±231 (220–1500) g (p=0.380). The mean±SD (min-max) birth weight was higher in live born (n=85) than stillborn (n=27) normal singletons infants: 863±235 [220–1500] and 652±208 [400–1320] g, (p<0.001). Mean birth weights for singletons and first-born twins were summarized for each gestational age week after excluding birth weight of those with congenital abnormality ( Supplementary File 2).

Congenital abnormality involved 10.6% (24/227, 30 missing) of exPTB and half of these congenital abnormality cases 50.0% (12/24) were stillborn ( Table 4).

**Table 4.** Congenital abnormalities, deformations and chromosomal abnormalities in 24 extreme preterm newborns, according to ICD-10 coding. ^a^multiple abnormalities, enalapril exposure; **also undiagnosed heart murmur.

System	Description	ICD-10 code	n
Central nervous system	Anencephaly	Q00.0	2
	Encephalocele ^a^	Q01	1
	Microcephaly	Q02	1
	Hydrocephalus	Q03	2
	Brain abnormality, unspecified	Q04.9	1
	Spina bifida	Q05	1
Cardiovascular	Malformation of cardiac chambers, not specified	Q20.9	1
Gastrointestinal	Oesophageal atresia	Q39	2
Urinary system	Outflow obstruction	Q64.7	1
Musculoskeletal	Talipes (home birth, few details)	Q66.4	1
	Phocomelia	Q72.2	2
Other	Fetal hydrops Not specified	P56	2
	Massive cystic hygroma	Q87.8	3
	Severe amniotic bands **	Q79.8	1
	Twin to twin transfusion syndrome	Q89.9	1
	(suspected) Trisomy-21	Q90	1

^a^Encephalocele and other abnormalities: arthryogyrophosis, micropthalmia – enalapril exposure

### Newborn survival

Of the 132 liveborn exPTB, the fate of one neonate was unknown at one month. Of the remaining neonates 98.5% (128/131) had a neonatal death and 1.5% (2/131) survived the first 28 days. The median [IQR, range] age of neonatal deaths was 1 [1–2, 1–28] day, with 87.0% (114/131) by day 3 ( Table 2). One newborn died at day 33 and the remaining child survived and was still alive when last seen at 40 months of age, with a normal neurodevelopment. The surviving female child was born at 27 ^+5^ weeks, and two ultrasounds, including an early scan at 8 weeks and a later scan at 18 weeks, assured the gestation. The mother was a 32-year-old refugee with a gravidity of two and parity of one, with no history of PTB, who went into spontaneous labour and received a single dose of nifedipine and dexamethasone less than one hour before delivery. Delivery was supervised by skilled birth attendants and after a normal vaginal birth of a 890g baby, the Apgar scores were six and seven, at one and five minutes, with no resuscitation necessary. The neonate was provided with supportive care (with oxygen delivered by nasal prongs; temperature control, phototherapy, breast milk) because that was all that was available, and discharged home after ten weeks at 1061g.

### The role of ultrasound

Use of ultrasound was associated with an increased adjusted odd ratio (AOR) of being classified as exPTB rather than miscarriage: AOR 2.09 (95%CI 1.31–3.34, p=0.002); while attending in the first trimester for the first antenatal visit and delivery at SMRU clinics were not associated: AOR 1.29 (95%CI 0.87–1.91, p=0.212), and AOR 0.93 (95%CI 0.61–1.45, p=0.802), respectively ( Table 5).

**Table 5.** The association between use of ultrasound and outcome classification as extreme PTB or miscarriage between 22 to <28 weeks (n=454). Numbers are % (n), Missing data: Place of birth SMRU (22). *Ultrasound was not introduced at SMRU until late 2001. Abbreviation: ANC antenatal consultation, SBA skilled birth attendant.

		Extreme PTB n=257	Miscarriage n=197	Odds Ratio (95% CI), P-value	Adjusted odds ratio (95% CI), p-value
Ultrasound *	Yes (all 2002–2015)	62.9 (207/125)	37.1 (122/329)	2.55 (1.67-3.88), p<0.001	2.04 (1.28-3.25), p=0.003
	No (all 1995–2001)	40.0 (50/329)	60.0 (75/125)		
Early ANC attendance	1st trimester	53.0 (133/251)	47.0 (118/251)	1.39 (0.96-2.03), p=0.084	1.30 (0.88-1.93), p=0.191
	2 ^nd^/3 ^rd^ trimester	61.1 (124/203)	38.9 (79/203)		
Delivery at clinic with SBA	Clinic	61.5 (177/287)	38.5 (110/267)	0.74 (0.50-1.12), p=0.151	0.93 (0.61-1.44), p=0.754
	Home	54.5 (79/145)	45.5 (66/145)		

## Discussion

Cautious interpretation of this data is required given the limitations of this dataset because 19% of women who registered to antenatal care had no pregnancy outcome reported. In a recent Lancet report of Myanmar Demographic Health Surveillance in 2016, less than 30% of rural women in Myanmar delivered in recognized institutions ^27^. In the context of this post-conflict, cross border, low resource setting, the fact that 81% of outcomes were reported, with ultrasound available for nearly ¾, supports the use of the data to bring clarity to the nature of the products of conception expulsed in the 22 to <28 weeks gestational age window. As well the median number of six antenatal consultations ( Table 1) is high for pregnancies ending before 28 weeks gestation in a limited-resource setting.

The window of gestation from 22 to <28 weeks involved less than 1% of all pregnancy outcomes and 4 in 10 outcomes were miscarriage (i.e. non-viable before reaching 22 weeks). Ultrasound was associated with a 2 times higher odds of the outcome being classified as an exPTB rather than a miscarriage. This suggests that ultrasound adds clarity to the outcome of pregnancy in this 22 to <28 week window, mostly because it detects the presence/absence of a fetal heart beat or in this setting even the presence of annovulatory pregnancy – both of which are not easily identified with more limited tools (pregnancy test, SFH and pinnard/hand held Doppler). Under the WHO/ICD 10 definition these miscarriages could be (wrongly) defined as exPTB based on gestation alone, which would falsely inflate stillbirth rates ^4^ ( Supplementary File 1).

Amongst the outcomes classified as exPTB, nearly one-half were stillborn, 98.5% of the babies born alive were neonatal deaths of which two-thirds occurred on day one, and there was a high proportion (as expected) of congenital abnormalities. A high congenital abnormality rate is expected amongst this age group in a setting that does not actively screen for abnormalities ^28^. Two infants emerged from the neonatal period, one died at day 33 and one female of 27+5 weeks gestation survived infancy (end of the first year). Not only are the outcomes of pregnancies of 22 to <28 weeks gestation in this setting dismal, the proportion of pregnancies that are involved, relative to all pregnancies with a known outcome, is small (0.9%). It could be argued that if more than palliative care was offered to the newborns at SMRU, outcomes may have been different but with no means to provide additional support such as intubation, incubators, surfactant, parenteral nutrition etc, improvements would remain marginal. The high proportion of stillbirths (47.6% (120/252) contrasts with rates under 2% previously reported in this population in the 28 to <34 week’ gestation window ^12^. Stillbirth rates may be inflated using gestational age rather than birthweight cut-offs ^29^ and also because nearly all these infants were born vaginally, including breech births, whereas caesarean section may have been offered in HIC. These different proportions are important because they indicate that in low resource setting, the maximum benefits of interventions to prevent newborn death, is in the group of 28 weeks (and above) gestation ^30^. Overall, this data supports the WHO definition of 28 weeks to define birth (live birth and stillbirth) ^31^, and <28 weeks as a pragmatic definition of miscarriage ^32^

In low resource settings there are many reasons why women may have a late outcome (22 to <28 weeks gestation) of a miscarriage (non-viable before 22 weeks) ( Table 3). Coming in to the clinic for an induction when there is no pain or bleeding (no obvious problem that is felt by the woman herself) may be perceived as being of greater consequence than not being able to plant or harvest crops, or not being able to receive daily wages which the family depend upon. Information on fetal movement was rarely volunteered so there may be cultural reasons for apparent tolerance to loss of fetal viability such as desire for *fetus papyraceus*, which, surprisingly, is culturally fortunate ^33^.

Registration of births is incomplete in many LIC ^34^ and dating pregnancies reliably a particularly challenging issue ^35^. Some of the higher end birth weights may be due to inaccurate dating but meta-analysis ^14^ also suggests 1000g as a cut-off misses gestations in the exPTB window. On the Thailand-Myanmar border, SMRU has integrated basic ultrasound delivered by local sonographers to routine ANC and this has been accepted by women ^36^. Efforts have been directed towards having local staff skilled in routine gestational age scanning ^23^, standard care at antenatal clinics and at birth ^15^, and in newborn care ^12^. Record keeping has been based on the cut-off point of 28 weeks gestation for birth and miscarriage and while that has been a strength in directing human resources in the delivery room and in special care baby unit to viable neonates it has also resulted in weaker reporting of pregnancy outcomes from 22 to <28 weeks, which is an obvious limitation of this data set. Another limitation of the analysis is the 21 year period of the cohort. This could however be viewed differently as it puts into perspective the small group of pregnancies involved: approximately six live born exPTBs per year compared to >2,000 births of 28 weeks or more per year. While there have been changes over time including the introduction of a special care baby unit and ultrasound, there has been no change in the assisted ventilatory support of newborns, which is not available.

In a low resource setting the outcome of pregnancy between 22 to <28 weeks gestation involves <1% of all outcomes, a high proportion of miscarriage (late expulsion), with one-in-two of the exPTBs being stillborn and amongst livebirths, a neonatal mortality approaching 100%. The distinction between miscarriage and exPTB in this gestational window is improved by ultrasound but this is unlikely to result in improvement in survival due to significant resource constraints. The WHO cut-point to define stillbirth and miscarriage is pragmatic and useful in low resource settings in the scale-up towards reducing preventable newborn deaths as it allows a greater focus on newborns more likely to survive with a gestation of 28 weeks or more.

## Data availability

Due to ethical and security considerations, the data that supports the findings in this study can be accessed only through the Data Access Committee at Mahidol Oxford Tropical Medicine Research Unit (MORU). The data sharing policy can be found here: http://www.tropmedres.ac/data-sharing. The application form for datasets under the custodianship of MORU Tropical Network can be found in Supplementary File 3.

## Notes

**Supplementary File 1**

Definitions of miscarriage and stillbirth: Internationally and at Shoklo Malaria Research Unit.

**Supplementary File 2**

Mean birth weight of extreme preterm births by gestational age between 22 to <28 weeks.

**Supplementary File 3**

Application form for data
