# Supplementary material for: The overlap between miscarriage and extreme preterm birth in a limited-resource setting on the Thailand-Myanmar border: a population cohort study
Source: Wellcome Open Res. 2018 Dec 6;1:32. Originally published 2016 Dec 23. [Version 3] doi: 10.12688/wellcomeopenres.10352.3 (PMC6305214; doi:10.12688/wellcomeopenres.10352.3)
Supplement: Supplementary file 1 [file wellcomeopenres-1-16304-s0002.tgz › ee73c186-d1fc-4dac-b3ab-574aacbd0fb4_Revised_supplementary_1.pdf]

Supplementary File 1: International Definitions for miscarriage vs stillbirth and live birth +/- neonatal death

Pregnancy Outcomes

Abortion / Miscarriage

Expulsion of embryo/fetus

WHO [1]
<500g or <**22weeks' gestation** / or <length 25 cm

USA (NCHS, CDC, ICD) [2]
<20 weeks' gestation / 350g

UK [3] **<24 weeks' gestation**

Stillbirth

"death prior to expulsion, irrespective of pregnancy duration; no signs life"

WHO/ICD-10 general [4,5]
≥500g / **22weeks'** gestation / length 25 cm
<u>Late fetal deaths</u> ≥1000 g, **≥28 weeks**
<u>Early fetal deaths</u>
500–1000g,
22–28 weeks

WHO International [4,5]
1000 g or **28 weeks or** 35 cm, or third-trimester

USA (CDC)[2]
≥20 weeks' gestation; ≥350g

UK [6]
≥24 weeks' gestation

Live Birth +/- Neonatal Death

Dependent on stillbirth definition

Live born +/- death 1st 28days

REFERENCES

1. WHO. Neonatal and perinatal mortality country, regional and global estimates. Geneva: World Health Organization; 2006.
2. Kowaleski J. State definitions and reporting requirements for live births, fetal deaths, and induced terminations of pregnancy (1997 revision). Hyattsville, MD: National Center for Health Statistics; 1997.
3. RCOG. Registration of stillbirths and certification for pregnancy loss before 24 weeks of gestation. UK: Royal College of Obstetricians & Gynaecologists; 2005.
4. WHO. International statistical classification of diseases and related health problems. - 10th revision, edition 2010. NLM classification: WB 15. Geneva: WHO; 2010.
5. Lawn JE, Gravett MG, Nunes TM, Rubens CE, Stanton C, Group GR. Global report on preterm birth and stillbirth (1 of 7): definitions, description of the burden and opportunities to improve data. BMC Pregnancy Childbirth. 2010;10 Suppl 1:S1.
6. Births and Deaths Registration (Northern Ireland) Order 1976, No. 1041 (N.I. 14) (1976).

SMRU Definitions for miscarriage vs stillbirth and live birth +/- neonatal death

Pregnancy Outcomes – SMRU publications to date (Nov 2018)

28 weeks' gestation separates miscarriage from birth and Neonatal Death

SMRU

Abortion / Miscarriage

<28 weeks' gestation

Fetal loss

SMRU

Stillbirth

≥28 weeks' gestation

Death prior expulsion, no signs of life (clinic-HR, home-no breaths/cry or

SMRU

Live Birth +/- Neonatal Death

≥28 weeks' gestation

Live born +/- death 1st 28days

Pregnancy Outcomes: SMRU this manuscript

Expulsed 22 to <28 weeks' gestation

This manuscript

Abortion/Miscarriage

Fetal **non-viability** confirmed
<22 weeks' gestation
BUT expulsed as fetal loss
22 to <28 weeks' gestation

This manuscript

Stillbirth

Fetal **viability** confirmed
22 to <28 weeks' gestation
AND expulsed as stillborn
22 to <28 weeks' gestation

Neonate with no signs of life (clinic-HR, home-no breaths/cry or movement)

This manuscript

Live Birth+/- Neonatal Death

Fetal **viability** confirmed
22 to <28 weeks' gestation
AND expulsed as live born
22 to <28 weeks' gestation

Live born +/- death 1st28days
