# Supplementary material for: The overlap between miscarriage and extreme preterm birth in a limited-resource setting on the Thailand-Myanmar border: a population cohort study
Source: Wellcome Open Res. 2018 Dec 6;1:32. Originally published 2016 Dec 23. [Version 3] doi: 10.12688/wellcomeopenres.10352.3 (PMC6305214; doi:10.12688/wellcomeopenres.10352.3)
Supplement: Supplementary file 2 [file wellcomeopenres-1-16304-s0000.tgz › edae76f0-6f44-4801-8216-d9ab475327c2.pdf]

Supplementary File 2. Mean birth weights for very preterm births 22 to <28 weeks gestation

		Weeks' gestation at pregnancy outcome					
	Total	22	23	24	25	26	27
Birth	257	16	21	39	40	71	60
Missing weight data	108/257 (42.0)	9/16 (56.3)	6/21 (28.6)	16/39 (41.0)	18/40 (45.0)	35/71 (49.3)	16/60 (26.7)
Congenital abnormality with weight	16/14 (10.7)	1/7 (1.5)	1/15 (6.7)	4/23 (17.4)	5/22 (22.7)	4/35 (11.4)	3/43 (7.0)
Singleton, Normal							
Available	112	6	13	18	14	27	34
Live birth	863±235 [220-1500] N=85	390±193, [220-600] n=3	578±133, [350-700], n=6	764±176 [555-1300] n=14	757±131 [510-1000] n=11	887±189 [500-1250] n=21	1036±173 [500-1500] n=30
Stillborn	652±208 [400-1320] N=27	513±12, [500-520] n=3	566±88, [440-680], n=7	633±106 [500-740] n=4	660±205 [460-870] n=3	713±327 [400-1320] n=6	830±233 [620-1060] n=4
Twins (1st born only), Normal							
Missing weight data	26	1	3	1	4	6	7
Available	17	0	1	1	3	5	7
Live birth	848±127 [600-1020] N=11	0	0	750 N=1	937±97 [830-1020] N=3	750±150 [600-900] N=3	879±102 [730-950] N=4
Stillborn	797±304 [500-1300] N=6	0	500 n=1	0	0	835±233 [670-1000] n=2	870±387 [550-1300] n=3
