# Supplementary material for: The overlap between miscarriage and extreme preterm birth in a limited-resource setting on the Thailand-Myanmar border: a population cohort study
Source: Wellcome Open Res. 2018 Dec 6;1:32. Originally published 2016 Dec 23. [Version 3] doi: 10.12688/wellcomeopenres.10352.3 (PMC6305214; doi:10.12688/wellcomeopenres.10352.3)
Supplement: Supplementary file 3 [file wellcomeopenres-1-16304-s0001.tgz › 34d8967f-c01d-4028-9acb-e6b279bca509.pdf]

Application Form for Datasets under the Custodianship of Mahidol Oxford Tropical Medicine Research Unit (MORU) Tropical Network

**1. APPLICANT DETAILS**				
**Name of applicant**				
**Institution name and address**				
**Phone**		**Email**		
**Name of applicant's supervisor/ manager/ head of department (delete as appropriate)**				
**Phone**		**Email**		
**2. DETAILS OF PROJECT**				
**Title of Project**			**Start Date**	
			**End Date**	
**3. CO-APPLICANTS INCLUDING MORU COLLABORATORS (if applicable)**				
**Name**	**Role**		**Institution**	
**Anticipated users or user groups other the above individuals e.g. data management team of applicant's institution**				
**4. BRIEF DESCRIPTION OF DATA REQUESTED**				
**5. OBJECTIVES OF PROJECT**				
**6. BRIEF DESCRIPTION OF ANALYSIS PLANNED**				
**7.POTENTIAL ETHICAL ISSUES INCLUDING RISKS e.g. stigmatisation or breaches of privacy**				
**8.POTENTIAL BENEFITS OF THE STUDY including to participant communities, scientific capacity building or health policy**				
**9. PLANNED OUTPUTS including publications**				

Signature of applicant: _____________ **DATE:** ______

Signature of applicant's supervisor/ manager/ head of department: _____________ **DATE:** _______

Note: Additional information may be requested to support the application. We are not charging for data access but the applicant may be required to cover the cost of preparing the data for sharing. Primary contact: phaikyeong@tropmedres.ac
