# Supplementary material for: The overlap between miscarriage and extreme preterm birth in a limited-resource setting on the Thailand-Myanmar border: a population cohort study
Source: Wellcome Open Res. 2018 Dec 6;1:32. Originally published 2016 Dec 23. [Version 3] doi: 10.12688/wellcomeopenres.10352.3 (PMC6305214; doi:10.12688/wellcomeopenres.10352.3)
Supplement: Peer review history

# Peer review: Referee response for version 3

**DOI:** 10.21956/wellcomeopenres.16304.r34388

**Published:** 2019-02-04

**Authors:** Halit Pinar

**Affiliations:** 1 Warren Alpert Medical School, Brown University, Providence, RI, USA

I reviewed the 3rd revision of the manuscript titled "Miscarriage, stillbirth and neonatal mortality in the extreme preterm birth window of gestation in a limited-resource setting on the Thailand-Myanmar border: a population cohort study’. I think the authors have addressed the concerns to the maximum of their capability within the confines of the study design and data set and I approve for publication.

I have read this submission. I believe that I have an appropriate level of expertise to confirm that it is of an acceptable scientific standard.

# Peer review: Referee response for version 3

**DOI:** 10.21956/wellcomeopenres.16304.r34409

**Published:** 2018-12-19

**Authors:** Glen D.L. Mola

**Affiliations:** 1 School of Medicine and Health Sciences, University of Papua New Guinea, Port Moresby, Papua New Guinea

The latest version of the paper "The overlap between miscarriage and extreme preterm birth in a limited-resource setting on the Thailand-Myanmar border; a population cohort study " version 3 is a significant improvement on previous 2 versions of this paper.

 However, I think it is worth mentioning that although it is sensible and logical to report as SBs those fetuses that are at least 28 weeks or 1000g, for international comparisons of PMRs, it is still worth gathering data for all fetuses (including SBs that are between 500-999g and/or  between 22-28 weeks gestation, - even though this paper is not about PMRs per se.)

I have read this submission. I believe that I have an appropriate level of expertise to confirm that it is of an acceptable scientific standard.

# Peer review: Referee response for version 2

**DOI:** 10.21956/wellcomeopenres.16004.r33532

**Published:** 2018-08-01

**Authors:** Waldemar A. Carlo

**Affiliations:** 1 Department of Pediatrics, The University of Alabama at Birmingham, Birmingham, AL, USA

**Abstract**

 The Methods section of the abstract does not distinguish well the classification used. It seems there were two groups but this is not clear from the structure of the sentence. How was non-viability versus viability assessed? 

 It is stated that non-viability was determined at a median GA of 16 weeks. It is unclear in what cases would non-viability of births at 22-28 weeks can be determined at 16 weeks.

**Methods**

 What was the quality control for data collection? Were clinicians trained in data collection? Were data collectors prospectively assessing the quality of the data? What QI efforts were made to improve data collection?

 Were the definitions of stillbirth versus neonatal death prospectively applied throughout the whole period of data collection? What signs of live birth were assessed? How was HR assessed?

 What resuscitation measures used in HR was not audible? Were data systematically collected on bag and mask ventilation, incubator use, IV fluids, oxygen supplementation, etc?

**Results**

 The outcome of 19% of booked pregnancies was not known. This is an important problem as pregnancies losses may be over-represented in these unknown outcome pregnancies.

 The stillbirth rate is very high especially at the lower gestations. For example, over 40% of births at up 26 weeks were assessed to be stillbirths. There is a frequent of limited assessment for live birth at the lowest gestations. It is common to assess that a newborn is stillbirth because resuscitation is not implemented when few signs of live birth are present. Signs of live birth are not reported. Resuscitation efforts are not reported.

 It is concerning that birth weight was not available for 42% of the births? Other basic data also were frequently not available. This raises concerns about the quality of the data.

 Gestational age may be underestimated as one SD is above 1000 g which is unlikely for a population of 22-27 week pregnancies.

 Data on provision of care are not provided well. It is unclear to what extent live births were provided full support (other than the therapies stated not to be available).

**Discussion**

 In the Discussion, the authors appear to implicate that palliative care was generally provided. Details on this should be provided in Results.

 The Discussion does not address most major limitations including the 19% of the pregnancies without outcome data, the post-natal interventions rate of use, and the rate of provision of palliative care.

 The Discussion is lengthy and yet does not address critical aspects of the limitations so the study can be pit in a better perspective and better interpret its contribution to the literature.

I have read this submission. I believe that I have an appropriate level of expertise to confirm that it is of an acceptable scientific standard, however I have significant reservations, as outlined above.

# Response

**Published:** 2018-11-27

**Authors:** Rose McGready

**Affiliations:** Shoklo Malaria Research Unit, Thailand

Referee Report Version 2

 01 Aug 2018 Waldemar A. Carlo, Department of Pediatrics, The University of Alabama at Birmingham, Birmingham, AL, USA 

 Approved with Reservations

 We would genuinely like to thank the reviewer Dr Waldemar A Carlo for his repeated review and acceptance to take time to understand this manuscript. The comments have helped to clarify the contents and limitations of the manuscript. A point – by – point response to each comment ( **in bold**) has been provided (below),

 Sincerely Rose

 On behalf of all authors

**Abstract**

**1.The Methods section of the abstract does not distinguish well the classification used. It seems there were two groups but this is not clear from the structure of the sentence. How was non-viability versus viability assessed?** 

 Amendments to abstract significant to correct this.

**2.It is stated that non-viability was determined at a median GA of 16 weeks. It is unclear in what cases would non-viability of births at 22-28 weeks can be determined at 16 weeks.**

 The sentence “Miscarriage (late expulsion) between 22 to <28 weeks was observed with non-viability occurring at an estimated median gestation of 16 weeks.” …says miscarriage – not birth: and has now been amended

 “Individual patient analysis of all miscarriages, estimated non-viability to have occurred at a median of 16 weeks despite late expulsion between 22 to <28 weeks.”

 Details are provided in the methods but the space limitation of the abstract prevents further elucidation of the reviewers query scenario e.g.

 A woman may present for the first time at 6 months (24 weeks GA) by LMP, she is sure of her dates but the ultrasound shows absent FHB in a fetus with a FL of 16 weeks (the HC may or may not be possible to obtain depending on why the fetus is so small) e.g FDIU since 16 weeks or IUGR only growing to a 16 week size. The expulsion occurs from 22 to <28 weeks pregnant based on LMP.  

**Methods**

**3.What was the quality control for data collection?**

 The records have been quality controlled for data entry since 1986 when the antenatal care program commenced, firstly by author FN and later by RM. The data has been quality controlled for completeness and correctness of entry as this has been the most intensely studied population (dataset) in terms of malaria in pregnancy worldwide.

 Gestational age has been a significant issue for decades as antimalarial drug exposures and pregnancy outcomes have been so important in this population where *P.falciparum* drug resistance is amongst the highest in the world. In addition significant efforts with gestational age were important to understand the association of artemisinin derivatives (the backbone of antimalarial treatment worldwide today) with fetal resorption.

**4.Were clinicians trained in data collection?**

 Yes

**5.Were data collectors prospectively assessing the quality of the data?**

 Yes

**6.What QI efforts were made to improve data collection?**

 Amendment at methods: section of data extraction

**7.Were the definitions of stillbirth versus neonatal death prospectively applied throughout the whole period of data collection?**

 Yes. See supplementary file 1 for clarification. The introduction has also been modified to conform with the reviewers suggestion.

**8.What signs of live birth were assessed?**

 In clinic based birth heart rate was assessed. In clinic and home births respiratory effort, and movement were assessed (or asked to the birth attendant).

**9.How was HR assessed?**

 Auscultation

**10.What resuscitation measures used in HR was not audible?**

 < 28 weeks none. This is not without reason in our experience: while the baby may live it does not live for very long as no accessory support can be provided.

 ≥ 28 weeks: 5 inflation breaths and then action dependent on HR according to the neonatal drill which is published elsewhere (Janet S et al **.** *PLoS One *2018, **13:**e0190419 which is ref 45 in v2..0).

 Clarified in the methods, in section “Setting”, last paragraph.

**11.Were data systematically collected on bag and mask ventilation, incubator use, IV fluids, oxygen supplementation, etc?**

 Yes, these data were systematically collected. We have clarified the text as follows:

 “If extreme PTB occurs (<28 weeks) infants are provided with palliative care. Parents are involved and counseled in the process ^10^ . No resuscitative efforts were offered to live born infants of 22 to <28 weeks’ gestation as no further sophisticated care i.e. intubation, incubators, surfactant, parenteral nutrition etc. was available, unlike infants of ≥28 weeks’ gestation.”[ref 45 in v2.0]

 The two references Turner ^10^ and Janet ^(ref 45 in version 2)^ (both open access) are important as they describe everything that does happen (e.g.bag and mask ventilation etc) when an infant is of 28 weeks’ gestation or more, in this low resource environment.

**Results**

**12.The outcome of 19% of booked pregnancies was not known. This is an important problem as pregnancies losses may be over-represented in these unknown outcome pregnancies.**

 We agree with the reviewer and we have added

- Amendment to the first sentence of the results (and abstract).
- This limitation of the data as the first paragraph to the discussion.

However we do not think that this limitation detracts from our main conclusion i.e. the need to focus on viable fetuses that have reached a GA of 28 weeks.

**13.The stillbirth rate is very high especially at the lower gestations. For example, over 40% of births at up 26 weeks were assessed to be stillbirths. There is a frequent of limited assessment for live birth at the lowest gestations. It is common to assess that a newborn is stillbirth because resuscitation is not implemented when few signs of live birth are present.**

 Thank you and this has been added as a limitation.

- the reviewers comment (Before the section on palliative care…)
- to further explain the high rate of stillbirth as many of these births would have been by emergency C.section in a HIC setting, not vaginal birth.(After the section on palliative care…)

 

**Signs of live birth are not reported.**

 Amended, see methods, data extraction and data definitions; 2 ^nd^ bullet point.

**Resuscitation efforts are not reported**.

 See point 11.

**14.It is concerning that birth weight was not available for 42% of the births? Other basic data also were frequently not available. This raises concerns about the quality of the data.**

 We agree this is a limitation. As the 28 week cut-point has been used to define birth and miscarriage it has been an ongoing process to motivate staff to weigh infants born prior to this gestation. Parents also see no reason to bring a stillborn or perinatal death infant to the clinic for a weight.

**15.Gestational age may be underestimated as one SD is above 1000 g which is unlikely for a population of 22-27 week pregnancies.**

 There are no SD above 1000g, only the upper range. These are possible from the local centiles produced for this population from ultrasound dated pregnancies (Rijken MJ et al. (2014) Quantifying Low Birth Weight, Preterm Birth and Small-for-Gestational-Age Effects of Malaria in Pregnancy: A Population Cohort Study. PLoS ONE 9(7): e100247. doi:10.1371/journal.pone.0100247). They may represent incorrect EGA but birthweight give lower stillbirth rates than gestational age cut-offs and this has been added to the discussion.

**14.Data on provision of care are not provided well. It is unclear to what extent live births were provided full support (other than the therapies stated not to be available).**

 Amended. This is the same as point 9. There was no resuscitation, intubation, incubators, surfactant, parenteral nutrition etc.

**Discussion**

**15.In the Discussion, the authors appear to implicate that palliative care was generally provided. Details on this should be provided in Results.**

 Amended as same as point 12.

**16.The Discussion does not address most major limitations including the 19% of the pregnancies without outcome data, the post-natal interventions rate of use, and the rate of provision of palliative care.**

 Amended as same as point 11 and 13.

 Limitations are addressed in paragraph 1, paragraph 3.

 In paragraph

**17.The Discussion is lengthy and yet does not address critical aspects of the limitations so the study can be pit in a better perspective and better interpret its contribution to the literature.**

 Agree: Significantly reduced

# Peer review: Referee response for version 1

**DOI:** 10.21956/wellcomeopenres.11154.r20881

**Published:** 2017-03-13

**Authors:** Shahirose Premji

**Affiliations:** 1 Faculty of Nursing, University of Calgary, Calgary, Canada

Thank you for the opportunity of reviewing this manuscript entitled “Miscarriage, stillbirth and neonatal mortality in the extreme preterm birth window of gestation in a limited-resource setting on the Thailand-Myanmar border: a population cohort study.” The work done is impressive and has the potential to provide insight in how we: (a) view and manage the ‘grey zone’ in low resource setting, (b) support mothers and their families who present for care when in this ‘grey zone’, and (c) should allocate the limited resources in reducing neonatal mortality in low resource setting.

**Title**

 The title is very long and needs to be shortened. Moreover, it needs to better capture the findings of the study.

**Abstract**

 Introduction: Clearly define WHO and study definitions – this is critical to understanding the study results. Objective: Associations were also examined (page 4) which is not captured here. Methods: a more comprehensive description is required. Results: please share the statistics related to the association found. Conclusion: bold and very relevant!

**Article Content:**

**Introduction**

 The definition of stillbirth, miscarriage, preterm birth, and neonatal death has been identified as problematic given overlaps in gestational cut-points. Hence, it is imperative that these terms be defined at the outset so that there is conceptual clarity both with respect to variables being examined and the intended purpose of the study. This would also help with interpretation of findings.

 There are discrepancies with respect to the definition of miscarriage in the introduction and methodology (e.g., bottom of page 4). Moreover, issues are highlighted about accuracy of gestational assessment, lack of ultrasound diagnosis of congenital abnormality which further impacts definitions of terms particularly with inclusion of data before 2001. It is therefore not clear what the study hopes to accomplish by examining pregnancy outcomes between 24 to < 28 weeks’ gestational age over a 20 year period.

    

 The second paragraph: the information pertaining to preterm births, neonatal deaths, and rationale for the high rates seems misplaced. Explain the relevance of the information shared to the aims of the study. Furthermore, explain the consequence(s) of poor reporting of stillbirth and neonatal deaths.

 The third paragraph: relevance of statistics related to mortality secondary to malaria is not clear. 

 The introduction should more clearly articulate the conceptual approach (i.e., operationalize definitions), explain the rationale of the study, and situate information related to preterm birth, neonatal deaths, maternal mortality within the context of study aims.

**Methodology**

**Data extraction and data definition**

 Data were reviewed from 1995 to 2015 which is problematic as care patterns have changed significantly in this time frame (details shared in second and third paragraph). For example, the authors explain that there has been a shift from home birth to hospital births and improvement in care during the antenatal period. These changes are likely to reduce fetal mortality, as well as maternal and neonatal mortality. Thus, it is not clear why this time span was used for the purpose of this study. Moreover, ultrasound was introduced in 2001 which means that accurate assessment of gestational age may be an issue prior to this time. Ultrasound has also changed the way in which loss of fetal viability was confirmed (e.g., fundal height measurements). Given the intent to describe viability and mortality in the window of 24 to <28 weeks’ gestational age it is important to have consistency in the way definitions are operationalized throughout the course of the study. 

 Data Quality – birth records are computer based; however is initial data recorded on paper and then transferred to the computer system? Please comment on the accuracy of data and strategies employed in ensuring quality data. Based on the results it appears that there are potential issues with data quality as in 4.4% of the 204 pregnancies between 24 and < 28 weeks’ gestational age there was missing data; birth weight data was available for 57.6% of neonates. This is a limitation and your discussion should explain how this impacts interpretation of findings.

 Ultrasound assessment – was this done by one person? If multiple assessors please comment on inter-rater reliability of ultrasound dating.

 Page 4 – please indicate the actual average of transporting women by car to the Thai hospital. Is this a potential limitation of the study?

**Statistical analysis**

 “Univariable and multivariable logistic regression was used to assess the association between estimation of gestational age by ultrasound, homebirth and year of birth, and birth outcome (birth rather than miscarriage).” A better description needs to be provided about the analytic plan and how it relates to the objectives of the study. The analytic plan should identify how variable were identified to be important to include in the multivariable logistic regression analysis. How were variables entered in the multivariable logistic regression analysis? Explain the rationale for the decision(s). It appears that the outcome of interest is birth however only 57.8% of these were live birth. What outcome was used? Furthermore, only one infant survived. What is the minimal sample size required for your multivariable regression analysis?

**Results**

 Pregnancy outcomes were unknown for 19.1% of the registered women – what was the issue?

 Only 50.6% of the women had their first antenatal care visit in trimester one which has implications for accuracy of gestational dating using ultrasound (most accurate in the first trimester).

 The inclusion of termination of pregnancy is confusing particularly since two were for maternal conditions at 24 and < 28 weeks’ gestation which in high income countries would be managed by inducing labour.

 Table 1 – Characteristic age, years – please indicate what the data represents (as you do for other variables).

 Figure 2 – study flow diagram can be expanded to more clearly display outcomes including stillbirths, live births, and survival.

 Figure 3 – Can be removed as I don’t think it provides any additional information.

**Discussion**

 The results do not demonstrate the bias detailed in the opening paragraph. Is there an opportunity here to compare historical cohorts (before introduction of ultrasound and after introduction of ultrasound)?

I have read this submission. I believe that I have an appropriate level of expertise to confirm that it is of an acceptable scientific standard, however I have significant reservations, as outlined above.

# Peer review: Referee response for version 1

**DOI:** 10.21956/wellcomeopenres.11154.r20244

**Published:** 2017-02-15

**Authors:** Halit Pinar

**Affiliations:** 1 Warren Alpert Medical School, Brown University, Providence, RI, USA

The study titled ‘Miscarriage, stillbirth and neonatal mortality in the extreme preterm birth window of gestation in a limited-resource setting on the Thailand-Myanmar border: a population cohort study’ is a well thought out and interesting study and has a lot of potential to be better and more useful to the reader.

 The following are some points I am hoping will be useful in the authors ’efforts to improve the manuscript.

**Recommendation 1:**

 The main objectives of the study are not clear. If it is challenging the definition (or lack thereof) of the miscarriages and its overlap with extreme premature deliveries, please say so more clearly. Obviously, this overlap is very confusing and just to demonstrate the conceptual difficulties one faces when trying to analyze a data set like this, is a worthy cause.

 Spend more time with these conceptual inconsistencies so the readers who are not familiar with these concepts will have a better understanding of your objectives and results.

**Recommendation 2:**

 Third paragraph in the Introduction:

 The background information given is interesting but very short. In addition, it is not clear what purpose it serves in the general context of the manuscript. Recommendation: It would be ideal to provide more detailed information that is pertinent to the etiology of maternal diseases, miscarriages, stillbirth etc. in that region to help the reader to better understand the characteristics of the patient population.

**Recommendation 3:**

 It appears there is a discrepancy between the definition of miscarriages at the end of the first paragraph of page 4 and the definition given under the header of miscarriage in the second column.

 The first definition: When a fetus dies <24 weeks but is delivered between 24-28 weeks.

 The second definition is used in the first sentence just under the subtitle of ‘miscarriage’: 114 women out of 318 women miscarried from 24<28 weeks’ gestation and the distribution is given according to the gestational age. In this section it appears that for a fetus to be called he/she does not have to die<24 weeks and be delivered after 28 weeks. In addition, in our experience it not very common for the mother to keep the dead fetus for two or more weeks (in this case up to 4 weeks).

**Recommendation 4:**

 Second column on page 6. The correct term for ‘surface’ examination is ‘external’ examination.

**Recommendation 5:**

 I would recommend exclusion of terminations. That adds more confusion to the calculations and it is considered a very different group.

**Question 1:**

 The true consequence of using different GA for the definition of fetal death/stillborn vs miscarriage (except causing confusion) is not clear.

**Question 2:**

 Since you have the numbers, what happens if you analyze the data where a pregnancy loss up to 20 weeks gestational age will be classified as a miscarriage and any delivery ≥20 weeks is classified either as a liveborn or stillborn?

I have read this submission. I believe that I have an appropriate level of expertise to confirm that it is of an acceptable scientific standard, however I have significant reservations, as outlined above.

# Response

**Published:** 2018-07-04

**Authors:** Rose McGready

**Affiliations:** Shoklo Malaria Research Unit, Thailand

**Halit Pinar, Warren Alpert Medical School, Brown University, Providence, RI, USA**

**Approved with Reservations**

**The study titled ‘Miscarriage, stillbirth and neonatal mortality in the extreme preterm birth window of gestation in a limited-resource setting on the Thailand-Myanmar border: a population cohort study’ is a well thought out and interesting study and has a lot of potential to be better and more useful to the reader.**

 Agree with potential to be better.

**The following are some points I am hoping will be useful in the authors ’efforts to improve the manuscript.**

**Recommendation 1:**

**The main objectives of the study are not clear. If it is challenging the definition (or lack thereof) of the miscarriages and its overlap with extreme premature deliveries, please say so more clearly. Obviously, this overlap is very confusing and just to demonstrate the conceptual difficulties one faces when trying to analyze a data set like this, is a worthy cause.**

 Thank you for your patience with the manuscript – we are trying to articulate i.e. that this window (22 to <28 weeks) can include miscarriage, a large proportion.

**Spend more time with these conceptual inconsistencies so the readers who are not familiar with these concepts will have a better understanding of your objectives and results.**

 We agree that we did not succeed to give clarity on the main objective. We have rephrased the entire manuscript. The aim of this study is to contribute to the sparse body of evidence from limited resource settings that support the use of the 28 weeks’ gestational age cut-point for stillbirth. We also argue for the usefulness of <28 weeks’ gestational age to define miscarriage.

**Recommendation 2:**

**Third paragraph in the Introduction:**

**The background information given is interesting but very short. In addition, it is not clear what purpose it serves in the general context of the manuscript. Recommendation: It would be ideal to provide more detailed information that is pertinent to the etiology of maternal diseases, miscarriages, stillbirth etc. in that region to help the reader to better understand the characteristics of the patient population.**

 Thank you for this comment – the introduction has been rewritten to address this point and Recommendation 1.

**Recommendation 3:**

**It appears there is a discrepancy between the definition of miscarriages at the end of the first paragraph of page 4 and the definition given under the header of miscarriage in the second column.**

**The first definition: When a fetus dies <24 weeks but is delivered between 24-28 weeks.**

**The second definition is used in the first sentence just under the subtitle of ‘miscarriage’: 114 women out of 318 women miscarried from 24<28 weeks’ gestation and the distribution is given according to the gestational age. In this section it appears that for a fetus to be called he/she does not have to die<24 weeks and be delivered after 28 weeks.**

 The definitions have been laid out more clearly. For this analysis the definitions of miscarriage is absence of viability before 22 weeks but delivery in the 22 to <28 week window and extreme preterm birth is presence of viability and delivery at 22 to <28 weeks.

**In addition, in our experience it not very common for the mother to keep the dead fetus for two or more weeks (in this case up to 4 weeks).**

 This is a very helpful comment and it is exactly what we want to highlight for an in issue relevant to low resource settings. We have an average of 6 weeks before expulsion.

 We suspect this must be a source of confusion in other low resource settings as these types of pregnancy outcomes are hard to document without a reliable method of measuring pregnancy viability e.g. ultrasound or multiple.

 We are also trying to highlight that the experience in low resource settings may not match the bulk of published evidence available.

**Recommendation 4:**

**Second column on page 6. The correct term for ‘surface’ examination is ‘external’ examination.**

 Amended as suggested

**Recommendation 5:**

**I would recommend exclusion of terminations. That adds more confusion to the calculations and it is considered a very different group.**

 Amended as suggested

**Question 1:**

**The true consequence of using different GA for the definition of fetal death/stillborn vs miscarriage (except causing confusion) is not clear.**

 We have spent more time to explain this in the introduction.

 The practice of obstetrics in resource limited settings is not as efficient. Miscarriage can occur at 22-28 weeks (the window of extreme preterm birth) and in the setting described in this manuscript where significant effort has been placed on assessment of gestational age, we present data to prove that point. The WHO definition of 28 week cut-point that defines a stillbirth is attractive and pragmatic for low resource settings.

**Question 2:**

**Since you have the numbers, what happens if you analyze the data where a pregnancy loss up to 20 weeks gestational age will be classified as a miscarriage and any delivery ≥20 weeks is classified either as a liveborn or stillborn?**

 We have gone back to the records to classify outcomes from 22 to <28 weeks gestation as miscarriage, livebirth, stillbirth, neonatal mortality. The records of 20 and 21 weeks start to have too much missing data to contribute reliably to the argument.

**I have read this submission. I believe that I have an appropriate level of expertise to confirm that it is of an acceptable scientific standard, however I have significant reservations, as outlined above. Competing Interests:** No competing interests were disclosed. Close
